# Economic outcomes associated with diagnosed behavioral symptoms among patients with dementia in the United States: a health care claims database analysis

**DOI:** 10.1186/s12877-023-03780-x

**Published:** 2023-02-17

**Authors:** Rezaul Karim Khandker, Farid Chekani, Kirti Mirchandani, Niranjan Kathe

**Affiliations:** 1 Center of Observational and Real-World Evidence, Merck & Co., Inc, 351 North Sumneytown Pike, North Wales, PA USA; 2 Complete HEOR Solutions (CHEORS), Chalfont, PA USA

**Keywords:** Dementia, Diagnosed behavioral symptoms, Economic burden, Health care resource utilization, Claims databases

## Abstract

**Background:**

Behavioral symptoms are common in patients with dementia. However, there is limited evidence of their economic burden. Among commercially insured patients with dementia in the United States, this study assessed the prevalence of diagnosed behavioral symptoms and whether healthcare resources utilization and costs were associated with these symptoms.

**Methods:**

This retrospective observational study was conducted using the IBM® MarketScan® Commercial Claims and Encounters and Medicare Supplemental database from October 1, 2015, to September 30, 2019. Diagnoses of dementia and behavioral symptoms were identified using the International Classification of Diseases, 10^th^ Modification codes. To test differences in patient characteristics among those with and without diagnosed behavioral symptoms, t-tests were used for continuous variables, and chi-square tests were used for categories. Generalized linear models were used to compare healthcare resource utilization and costs between patients with and without diagnosed behavioral symptoms, adjusted for baseline characteristics.

**Results:**

Of the 62,901 patients with dementia included in the analysis, 16.5% had diagnosed behavioral symptoms 12 months post dementia diagnosis. Patients with diagnosed behavioral symptoms used more health care resources (mean annual pharmacy visits per patient: 39.83 vs. 33.08, mean annual outpatient visits per patient: 24.20 vs. 16.94, mean annual inpatient visits per patient: 0.98 vs. 0.47, mean annual ER visits per patient: 2.45 vs. 1.21) and incurred higher cost of care than those without diagnosed behavioral symptoms (mean annual total health care costs per patients: $63,268 versus $33,383). Inpatient care was the most significant contributor to total costs (adjusted annual mean cost per patient: $28,195 versus $12,275).

**Conclusion:**

Behavioral symptoms were significantly associated with higher healthcare resource utilization and costs among patients with dementia. Further research is warranted to address the unmet medical needs of this patient population.

**Supplementary Information:**

The online version contains supplementary material available at 10.1186/s12877-023-03780-x.

## Background

Dementia, often regarded as a chronic disease associated with aging, hinders the independent functioning and cognitive abilities of patients [1]. Symptoms are accompanied by emotional disturbances and personality changes ranging in severity from the mildest stage, when it is just beginning to affect a person’s functioning, to the most severe stage, when the person must depend entirely on others for basic activities of living [2]. Patients with dementia, due to organic diseases or disorders of the brain, experience deterioration of their intellectual faculties. Alzheimer’s disease is the most common cause of progressive dementia in older adults, accounting for 60–70% of cases [3]. However, there are several other types of dementia, including vascular dementia, frontotemporal dementia, Lewy body dementia, or a combination of several types referred to as mixed dementia [3]. With the aging of the global population, an increasing number of individuals suffer from this condition. It is expected to increase from approximately 57.4 million cases in 2019 to 152.8 million cases in 2050 [4]. Dementia has significant social and economic implications regarding direct medical and social care costs and informal care. In 2015, the total global societal cost of dementia was estimated at US$ 818 billion, equivalent to 1.1% of the global gross domestic product [5].

Behavioral and psychological symptoms of dementia, also referred to as neuropsychiatric symptoms, often accompany cognitive impairment. These include psychotic symptoms such as delusions and hallucinations, expansive symptoms such as aggression, irritability, nighttime disturbances, aberrant motor behavior, affective symptoms such as anxiety, apathy, and depression, and other symptoms such as disinhibition euphoria [6]. Irrespective of the underlying disease, behavioral and psychological symptoms constitute a significant clinical burden, resulting in substantial morbidity and caregiver burden. They also strongly correlate with the degree of functional and cognitive impairment and determine a patient’s lifestyle and management [7, 8]. For instance, compared with other patients with dementia, patients with agitation experience faster cognitive and functional decline [9], more rapid disease progression [10, 11], and earlier death [11]. Behavioral and psychological symptoms affect nearly all patients living with dementia at some point during their disease [3, 8, 12] and the consequences can be devastating [8, 13–15]. These symptoms can be highly stressful for the patient and their caregivers, affecting their quality of life and representing significant reasons for the institutionalization of dementia patients [6, 8, 12, 14–19].

To date, no medication has been approved by the United States (U.S.) Food and Drug Administration for managing behavioral symptoms in patients with dementia. In clinical practice, the symptoms are frequently managed with antipsychotics, antidepressants, and antiepileptics. Benzodiazepines are also over-utilized despite their demonstrated risk for harm and poor efficacy.

Real-world evidence regarding the economic burden of behavioral symptoms in patients is needed to estimate the value of novel treatments for patients with dementia. Claims data sources provide rich information on clinical and treatment-related characteristics, healthcare utilization, and costs. Few studies have examined the health care resource utilization (HCRU) and costs associated with behavioral symptoms, given the underdiagnosis and undertreatment of such symptoms among patients with dementia [18, 20–22]. Therefore, in this study, we leverage one of the largest sources of claims data to fill this gap in the literature. This study aims to characterize the prevalence of diagnosed behavioral symptoms among commercially insured older adults with dementia in the U.S. and to assess the association of these symptoms with HCRU and related costs.

## Methods

### Study design and data source

**Fig. 1:**
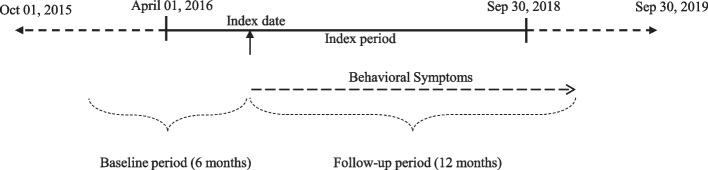
Study design

This retrospective observational study was conducted using the IBM® MarketScan® Commercial Claims and Encounters and Medicare Supplemental Database. The database includes inpatient, outpatient, pharmacy claims, and enrollment details from more than 40 million employees, their spouses, and dependents covered by private health care insurance with or without Medicare supplemental coverage. The study design is illustrated in Fig. 1. The study used medical and pharmacy claims recorded during the study period from October 1, 2015, when the transition from the International Classification of Disease (ICD) ninth revision to the tenth version occurred, to September 30, 2019, as this was the most recent period available in the databases. The analysis included adult patients aged 65 years and above with at least one inpatient or two outpatient diagnoses of dementia during the index period going from April 1, 2016, to September 30, 2018. The first date of dementia diagnosis during the index period was defined as the index date. A prevalent cohort of patients with dementia was utilized as these patients reflect a more generalizable population.

Patients were excluded if they had an enrollment gap greater than 45 days in pharmacy, medical, or mental health coverage 6 months prior (baseline) or 12 months after the index date. Patients with a history of schizophrenia (ICD-10 = F20.XX, F250, F251, F258, F259) or bipolar disorder (ICD-10 = F31.XX) in the baseline period were excluded since the symptoms may overlap with diagnosed behavioral symptoms in dementia.

Dementia and behavioral symptoms were identified through the ICD-10 codes (Refer to Supplementary Table 1 for ICD-10 codes for dementia). Behavioral symptoms were defined as the presence of any symptoms of agitation/aggression, psychosis, or delirium/wandering (Refer to Supplementary Table 2 for ICD-10 categories for each behavioral symptom) at any position including primary diagnosis (PDX), DX1-DX5.

The prevalence of diagnosed behavioral symptoms, HCRU, and costs were measured during the 12-month post-dementia diagnosis. HCRU and costs were compared between patients with and without diagnosed behavioral symptoms post dementia diagnosis.

### Study measurements

Demographics were measured at the index date, and comorbidities and drug utilization were determined during the 6-month baseline period. The prevalence of behavioral symptoms and specifically, of agitation/aggression, psychosis, and delirium/wandering, was obtained by dividing the number of patients with at least one diagnosis during the 12-month post dementia diagnosis by the total number of patients included in the analysis.

The following types of medical services were counted for each patient: inpatient visits (acute and non-acute, i.e., long-term care), inpatient days of stay, emergency room (ER) visits, physician office visits, outpatient visits, other medical claims (including other outpatient service claims and hospice visits), and pharmacy visits (distinct days with at least one pharmacy claim).

HCRU costs were calculated as the sum of the health plan paid and patient out-of-pocket expenses (co-insurance, copayment, and deductibles). Health plan paid costs included any coordination of benefits or other savings. Costs were adjusted to 2020 U.S. dollars using the consumer price index medical care component. The total health care costs were the sum of medical care costs and pharmacy costs.

### Statistical analysis

Descriptive statistics were used, and significance tests were conducted to assess the differences between patients with and without diagnosed behavioral symptoms. Continuous variables were evaluated using t-tests, and categorical variables were evaluated using chi-squared tests.

Separate multivariable generalized linear models were used to compare each HCRU and cost outcomes between patients with and without diagnosed behavioral symptoms to account for the differences in covariates. In each model, the baseline covariates included were age group, index year, gender, insurance plan type, region of residence, urbanicity, use of antidepressants, use of antipsychotics, use of ASH benzodiazepines, use of Anxiolytic/Sedative/Hypnotic not elsewhere classified, the Elixhauser Comorbidity Index score, and institutionalization, as well as the each of the following comorbidities: hypertension, diabetes, cancer, anemia, congestive heart failure, cardiac arrhythmia, chronic pulmonary disease, renal failure, fluid and electrolyte disorders, and depression.

For outcomes where more than 30% of the patients had a value of zero, a two-part model was used instead of a one-part model. The first part of the two-part model estimated the odds ratios (OR) of having diagnosed behavioral symptoms compared to those without, and the second part estimated the incidence rate ratio (IRR) within the subgroup of patients with a positive outcome, for example, patients with at least one inpatient visit. With the one-part models, the IRR reflects the outcomes in all patients. For costs, the models used the gamma distribution. For HCRU, the models used either Poisson, negative binomial, or gamma distribution which was selected based on the Akaike Information Criteria. Statistical significance was based on an alpha level of 5%. Additionally, marginal effect statistics were calculated for two-part HCRU and costs categories using the recycled predictions technique.

## Results

### Construction of the study sample

There were 151,626 commercially insured patients aged 18 years old and above with at least one inpatient claim or at least two outpatient claims for dementia during the index period, of whom 118,216 had continuous coverage for the 6-month baseline period. From this group, 70,427 patients had continuous coverage in the database for 12 months post dementia diagnosis, of whom 1,983 were excluded given a diagnosis of schizophrenia or bipolar disorder during the baseline period. Of the remaining 68,444 patients, 62,901 were aged at least 65 years old and were included in the analysis. For 35,500 of the 62,901 patients with dementia included in the analysis (56.4%), the diagnosis was “unspecified dementia.” Of the 62,901 patients included, 16,927 (26.9%) had a diagnosis of Alzheimer's disease.

### Prevalence of diagnosed behavioral symptoms

The prevalence of any diagnosed behavioral symptoms during the 12-month post-dementia diagnosis was 16.5% among all dementia patients versus 13.6% among patients diagnosed with Alzheimer's disease. Among all patients, the prevalence of agitation and aggression, psychosis, delirium, and wandering were 5.0%, 9.1%, and 6.3%, respectively. Among patients with Alzheimer's disease, the prevalence of agitation and aggression, psychosis, delirium, and wandering over the 12 months post dementia diagnosis was 4.4%, 7.4%, and 4.7%, respectively.

### Baseline demographics and clinical characteristics

Comorbidities were more frequent among patients diagnosed with behavioral symptoms during the 12-month post-dementia diagnosis compared to those who were not, whereas the other baseline characteristics were similar between the two groups (Table 1). Patients were aged 84 years old on average, and 61.7% were female.

**Table 1:** Baseline characteristics of patients with and without diagnosed behavioral symptoms 12 months post dementia diagnosis

Baseline Characteristics	With diagnosed behavioral symptoms *N* = 10,408	Without diagnosed behavioral symptoms *N* = 52,493	*P*-value
**Age**			
Mean (SD)	84.4 (7.6)	84.3 (7.6)	0.3092
**Age-group N (%)**			
65–69	367 (3.5)	1,971 (3.7)	0.4396
70–74	821 (7.9)	4,080 (7.8)	
75–79	1,485 (14.3)	7,486 (14.3)	
80–84	2,232 (21.4)	11,582 (22.1)	
85 +	5,503 (52.9)	27,374 (52.1)	
**Index Year N (%)**			
2016	7,509 (72.2)	38,669 (73.7)	**0.0008**
2017	2,073 (19.9)	10,129 (19.3)	
2018	826 (7.9)	3,695 (7.0)	
**Gender N (%)**			
Female	6,253 (60.1)	32,547 (62.0)	**0.0002**
Male	4,155 (39.9)	19,946 (38.0)	
**Plan Type N (%)**			
Comprehensive	5,503 (52.9)	27,040 (51.5)	** < .0001**
Exclusive Provider Organization	2 (0.0)	22 (0.0)	
Health Maintenance Organization	409 (3.9)	2,193 (4.2)	
Point of service	146 (1.4)	855 (1.6)	
Preferred Provider Organizations	4,010 (38.5)	20,298 (38.7)	
Point of service with capitation	165 (1.6)	1,241 (2.4)	
Consumer-driven health plan	44 (0.4)	270 (0.5)	
High deductible health plan	6 (0.1)	58 (0.1)	
Unknown (Missing)	123 (1.2)	516 (1.0)	
**Urbanicity N (%)**			
Rural	2,629 (25.3)	13,576 (25.9)	0.2020
Urban	7,779 (74.7)	38,917 (74.1)	
**Geographic Region N (%)**			
Northeast	2,972 (28.5)	14,250 (27.1)	** < .0001**
North Central	3,690 (35.4)	17,474 (33.3)	
South	2,913 (28.0)	15,929 (30.3)	
West	822 (7.9)	4,778 (9.1)	
Unknown	11 (0.1)	62 (0.1)	
**Clinical Characteristics**			
**Elixhauser Index Score**			
Mean (SD)	5.4 (4.7)	4.6 (4.4)	** < .0001**
**Comorbidities in the baseline period N (%)**			
Congestive Heart Failure	1,734 (16.7)	7,259 (13.8)	** < .0001**
Cardiac Arrhythmia	3,119 (30.0)	13,623 (26.0)	** < .0001**
Valvular Disease	1,287 (12.4)	5,666 (10.8)	** < .0001**
Pulmonary Circulation Disorders	353 (3.4)	1,450 (2.8)	**0.0006**
Peripheral Vascular Disorders	2,417 (23.2)	10,051 (19.1)	** < .0001**
Hypertension Uncomplicated	6,920 (66.5)	32,477 (61.9)	** < .0001**
Hypertension Complicated	1,232 (11.8)	5,116 (9.7)	** < .0001**
Paralysis	258 (2.5)	943 (1.8)	** < .0001**
Other Neurological Disorders	2,985 (28.7)	11,215 (21.4)	** < .0001**
Chronic Pulmonary Disease	1,846 (17.7)	8,072 (15.4)	** < .0001**
Diabetes Uncomplicated	2,506 (24.1)	11,489 (21.9)	** < .0001**
Diabetes Complicated	1,694 (16.3)	7,880 (15)	**0.0011**
Hypothyroidism	1,839 (17.7)	8,682 (16.5)	**0.0051**
Renal Failure	1,461 (14.0)	6,382 (12.2)	** < .0001**
Liver Disease	208 (2.0)	908 (1.7)	0.0615
Peptic Ulcer Disease, excluding bleeding	85 (0.8)	336 (0.6)	**0.0482**
AIDS/HIV	6 (0.1)	32 (0.1)	1.0000
Lymphoma	71 (0.7)	457 (0.9)	0.0596
Metastatic Cancer	83 (0.8)	374 (0.7)	0.3436
Solid Tumor without Metastasis	810 (7.8)	3,903 (7.4)	0.2214
Rheumatoid Arthritis/collagen	366 (3.5)	1,772 (3.4)	0.4773
Coagulopathy	375 (3.6)	1,503 (2.9)	** < .0001**
Obesity	415 (4.0)	1,927 (3.7)	0.1192
Weight Loss	824 (7.9)	2,984 (5.7)	** < .0001**
Fluid and Electrolyte Disorders	2,000 (19.2)	6,922 (13.2)	** < .0001**
Blood Loss Anemia	173 (1.7)	684 (1.3)	**0.0047**
Deficiency Anemia	783 (7.5)	3,130 (6.0)	** < .0001**
Alcohol Abuse	178 (1.7)	525 (1.0)	** < .0001**
Drug Abuse	91 (0.9)	299 (0.6)	**0.0006**
Depression	2,743 (26.4)	9,237 (17.6)	** < .0001**
**Medications N (%)**			
Antidepressant Medications	4829 (46.4)	20,959 (39.9)	** < .0001**
Antipsychotic Medications	2397 (23.0)	5173 (9.9)	** < .0001**
ASH/Benzodiazepines	1882 (18.1)	6250 (11.9)	** < .0001**
ASH not elsewhere classified	605 (5.8)	2,365 (4.5)	** < .0001**

Continuous variables were evaluated using t-tests, and categorical variables were evaluated using chi-squared tests. *P*-values in bold format are the significantly different variables (< 5% level of significance)

*ASH* Anxiolytic, sedative, and hypnotic, *SD* standard deviation

### Comparison of HCRU and costs between patients with and without diagnosed behavioral symptoms

The results from the multivariate models suggest that patients with diagnosed behavioral symptoms utilize more health care resources than patients without diagnosed behavioral symptoms (Table 2). Patients with diagnosed behavioral symptoms were 231% more likely to have an inpatient visit (OR [95% CI]: 3.31 [3.16, 3.46]), and among those who had at least one inpatient visit, the number of inpatient visits was 13% higher (IRR[95%CI]: 1.13 [1.11, 1.16]. Similarly, patients with diagnosed behavioral symptoms were 176% more likely to have an ER visit (OR [95% CI]: 2.76 [2.63, 2.89]), and among those who had at least one visit, the number of visits was higher by 33% (IRR [95% CI]: 1.33 [1.30, 1.35]). Patients with diagnosed behavioral symptoms also had 35% more outpatient visits (IRR [95% CI]: 1.35 [1.33, 1.37]), 90% more outpatient claims (IRR [95% CI]: 1.90 [1.85, 1.96]), and 10% more pharmacy visits (IRR [95% CI]: 1.10 [1.08, 1.11]) than those without diagnosed behavioral symptoms. Compared to other patients, those diagnosed with behavioral symptoms were 5% less likely to have a physician’s office visit (OR [95% CI]: 0.95 [0.91, 1.00]), and among those who had at least one visit, the number of physician office visits was 6% lower (IRR [95% CI]: 0.94 [0.92, 0.97]). The results in terms of costs were concordant. However, the costs of pharmacy visits were lower among patients with diagnosed behavioral symptoms (IRR [95% CI]:0.97 [0.94, 1.00]). Overall, when considering baseline covariates, the total cost was higher in patients with diagnosed behavioral symptoms than in those without diagnosed behavioral symptoms (IRR [95% CI]:1.75 [1.72, 1.79]).

**Table 2:** Comparison of HCRU and costs between patients with and without diagnosed behavioral symptoms 12-month post-dementia diagnosis using multivariate regression models

HCRU component	OR [95% CI] for counts ≥ 0	IRR [95% CI]^a^	Cost component	OR [95% CI] for costs ≥ 0	IRR [95% CI]^a^
Inpatient days of stay	3.31 [3.16, 3.46]	1.48 [1.42, 1.56]	Inpatient	3.31 [3.16, 3.46]	1.24 [1.21, 1.27]
Inpatient visits	3.31 [3.16, 3.46]	1.13 [1.11, 1.16]	ER	2.75 [2.63, 2.89]	1.23 [1.20, 1.27]
ER visits	2.76 [2.63, 2.89]	1.33 [1.30, 1.35]	Physician office	0.95 [0.91, 1.00]	0.89 [0.85, 0.92]
Physician office visits	0.95 [0.91, 1.00]	0.94 [0.92,0.97])	Outpatient	One-part model	1.19 [1.17, 1.22]
Outpatient visits	One-part model	1.35 [1.33, 1.37]	Other medical claims b	One-part model	2.02 [1.93, 2.12]
Other medical claims b	One-part model	1.90 [1.85, 1.96]	Pharmacy	One-part model	0.97 [0.94, 1.00]
Pharmacy visits	One-part model	1.10 [1.08, 1.11]	Total	One-part model	1.75 [1.72, 1.79]

OR and IRR are for patients with diagnosed behavioral symptoms relative to those without diagnosed behavioral symptoms

*ASH* Anxiolytic, sedative, and hypnotic, *CI* Confidence interval, *IRR* Incidence rate ratio, *OR* Odds ratio

^a^For the one-part models, the IRR reflects the outcomes in all patients as opposed to only those with an outcome value above zero. In each model, the baseline covariates included were age group, index year, gender, insurance plan type, region of residence, urbanicity, use of antidepressants, use of antipsychotics, use of ASH/benzodiazepines, use of ASH not elsewhere classified, the Elixhauser Index Score, and institutionalization, as well as the each of the following comorbidities: hypertension, diabetes, cancer, anemia, congestive heart failure, cardiac arrhythmia, chronic pulmonary disease, renal failure, fluid and electrolyte disorders, and depression

^b^Other medical claims combine hospice-related visits and claims classified as “other outpatient service”

**Fig. 2:**
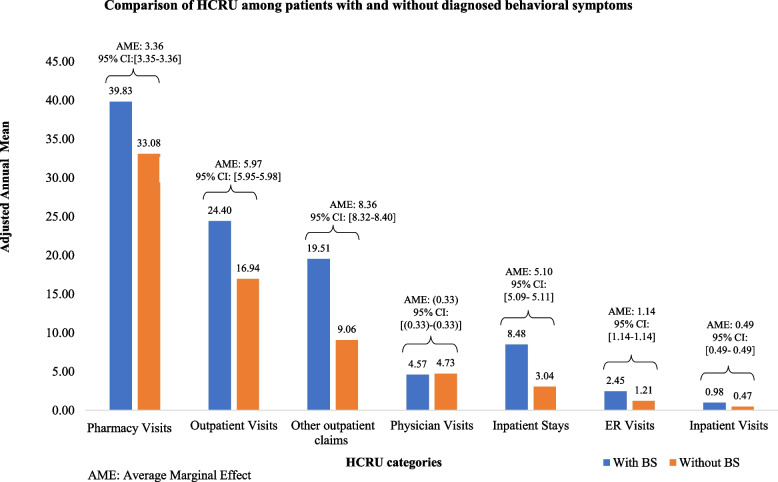
Comparison of adjusted annual mean HCRU per patient between patients with and without diagnosed behavioral symptoms. Adjusted annual means were obtained from one-part (outpatient visits, other medical claims, and pharmacy visits) or two-part (inpatient visits, inpatients days, ER visits, and physician office visits) multivariate generalized linear models. In the two-part models, the odds ratios for a positive value were obtained in the first part of the model, and for the second part, the counts were modelled within the subgroup of patients with a positive outcome. With the one-part model, the counts were modelled in all patients. The models used a distribution selected between Poisson, negative binomial, and gamma based on the Akaike information criteria. In each model, the baseline covariates included were age group, index year, gender, insurance plan type, region of residence, urbanicity, use of antidepressants, use of antipsychotics, use of ASH/benzodiazepines, use of ASH not elsewhere classified, the Elixhauser Index Score, and institutionalization, as well as the each of the following comorbidities: hypertension, diabetes, cancer, anemia, congestive heart failure, cardiac arrhythmia, chronic pulmonary disease, renal failure, fluid and electrolyte disorders, and depression. The average marginal effects represent, in absolute terms, the incremental counts associated with behavioral symptoms when all other covariates are kept constant, using a recycled prediction technique. The mean number of inpatient days of stay per patient is calculated for patients with at least one inpatient visit. ASH: Anxiolytic, sedative, and hypnotic; BS: Behavioral Symptoms; CI: confidence interval; ER: the emergency room

The adjusted per patient mean annual all-cause utilization of the different health care resource components during the 12-month post-dementia diagnosis, with and without diagnosed behavioral symptoms, is depicted in Fig. 2. Except for physician office visits, the utilization of health care resources was higher among patients diagnosed with behavioral symptoms than those without. For example, the adjusted annual mean number of inpatient visits was 0.98 and 0.47 among patients with and without diagnosed behavioral symptoms, respectively. The adjusted annual mean number of ER visits was 2.45 in patients diagnosed with behavioral symptoms versus 1.21 among those who were not.

**Fig. 3:**
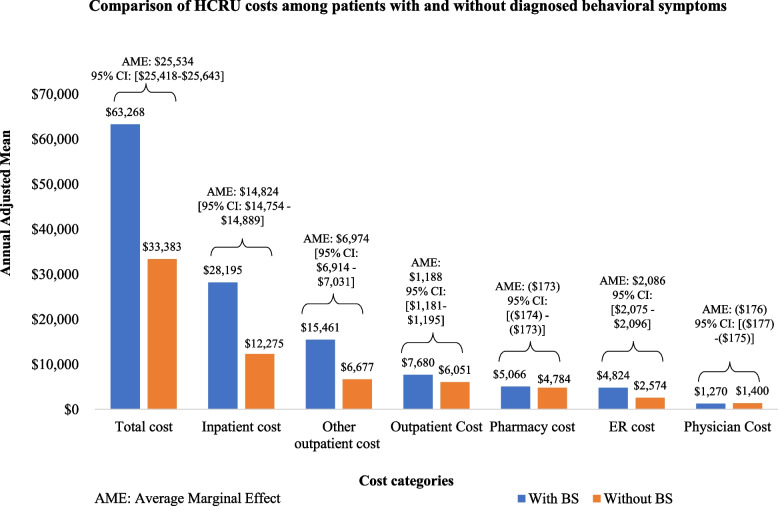
Comparison of adjusted annual mean costs per patient between patients with and without diagnosed behavioral symptoms. Adjusted annual means were obtained from one-part (outpatient, other medical claims, pharmacy, and total costs) or two-part (inpatient, ER, physician office) multivariate generalized linear model. In the two-part models, the odds ratios of a positive value were obtained on the one hand, and the cost was modelled within the subgroup of patients with a cost greater than zero. With the one-part model, the cost was model for all patients. In both cases, the models were based on a gamma distribution. In each model, the baseline covariates included were age group, index year, gender, insurance plan type, region of residence, urbanicity, use of antidepressants, use of antipsychotics, use of ASH/benzodiazepines, use of ASH not elsewhere classified, the Elixhauser Index Score, and institutionalization, as well as the each of the following comorbidities: hypertension, diabetes, cancer, anemia, congestive heart failure, cardiac arrhythmia, chronic pulmonary disease, renal failure, fluid and electrolyte disorders, and depression. The average marginal effects represent, in absolute terms, the incremental cost associated with behavioral symptoms when all other covariates are kept constant, using a recycled prediction technique. Costs were adjusted to 2020 U.S. dollars using the medical care component of the Consumer Price Index. ASH: Anxiolytic, sedative, and hypnotic; BS: Behavioral Symptoms; CI: confidence interval; ER: emergency room

Figure 3 presents the adjusted mean annual all-cause costs per patient during the 12-month post-dementia diagnosis for each health care component, with and without diagnosed behavioral symptoms. Total costs were higher for patients with than for patients without diagnosed behavioral symptoms (adjusted annual mean cost per patient: $63,268 versus $33,383). Most of these costs were attributable to medical claims (the adjusted mean annual pharmacy costs for patients with and without diagnosed behavioral symptoms were $5,066 and $4,784, respectively). Excluding physician office visits, each medical care cost component was higher for patients with diagnosed behavioral symptoms than for other patients. Inpatient care was the most significant contributor to total costs (adjusted annual mean cost per patient: $28,195 versus $12,275), followed by the costs incurred by other medical claims (adjusted annual mean cost per patient: $15,461 versus $6,677) and by outpatient costs (adjusted annual mean cost per patient: $7,680 versus $6,051).

Keeping other covariates constant, patients diagnosed with behavioral symptoms 12 months post dementia diagnosis were associated with 3.36 additional pharmacy visits, 5.97 additional outpatient visits, 8.36 additional other medical claims, 0.33 fewer physician visits, 5.10 additional inpatient days, 1.14 additional ER visits, and 0.49 additional inpatient visit (Fig. 2). With respect to costs, having behavioral symptoms was associated with incremental total costs of $25,534, incremental inpatient costs of $14,824, the incremental cost for other medical claims of $6,974, incremental outpatient costs of $1,188, incremental pharmacy costs of ($173), incremental ER costs of $2086, incremental physician visit costs of ($176) (Fig. 3).

## Discussion

With an aging population, dementia will affect an increasing proportion of individuals across the U.S., and many of these patients will also experience behavioral symptoms. We conducted a retrospective analysis using a health care claims database. We evaluated the prevalence of diagnosed behavioral symptoms in commercially insured older adults with dementia from the U.S. Multivariable regressions were used to compare HCRU and related costs between patients with and without diagnosed behavioral symptoms over 12 months while adjusting for patient characteristics, including demographics, drug utilization, and comorbidities.

In this sample of people with dementia aged 65 years and above, the claims-based prevalence of diagnosed behavioral symptoms was estimated to be 17% over 12 months and was similar (14%) in the subgroup of patients with Alzheimer's disease. In contrast, the literature suggests that these symptoms are highly prevalent, even in the early stages of cognitive impairment, and that virtually all patients experience behavioral symptoms at some point throughout their illness. The prevalence has previously been reported to be 90% throughout the illness [8, 12], and in patients with mild cognitive impairment, estimates vary from 35 to 85%. [23]. Also, Chekani et al. recently estimated the prevalence of behavioral symptoms to be approximately 81% among dementia patients using data from 2015/16 Adelphi Real World Dementia Disease-Specific Programme™ [19]. The low prevalence in our study may be attributed to the difficulty in identifying behavioral symptoms using claims data, highlighting the underdiagnosis, underreporting, and undertreatment of these symptoms among patients with dementia. Moreover, these symptoms are generally under-coded in ambulatory visits. The cohort of dementia patients with Commercial and Medicare supplemental coverage is different from those who reside in long-term care facilities. Therefore, this study might have underestimated the prevalence of behavioral symptoms in dementia patients. Provider education and awareness programs should be designed to help the providers diagnose the presence of behavioral symptoms among patients with dementia and initiate the management and treatment for the same. The low prevalence of diagnosed behavioral symptoms is concerning and highlights the need to for additional research on developing and improving algorithms for the identification of behavioral symptoms among patients with dementia using secondary databases. Moreover, future research should focus on identifying behavioral symptoms through medication use, including antipsychotics, antidepressants, antiepileptics, anxiolytics, etc., and compare cost associated with medication use versus without medication use among patients with dementia and behavioral symptoms.

The regression models accounting for various covariates revealed that patients with diagnosed behavioral symptoms used more healthcare resources and incurred higher healthcare costs than those without diagnosed behavioral symptoms. This corroborates Eddie et al.'s recent findings, which showed, using data from the 2015/16 Adelphi Real World Dementia Disease-Specific Programme™, a positive association between agitation in dementia and HCRU and health care cost [21]. Our results also align with those of another study by Aigbogun et al., who used MarketScan® claims data to assess the association between behavioral disturbances and HCRU and costs in specific types of dementia. Their findings were similar to ours, with 0.97 versus 0.62 annual adjusted per-patient hospitalizations in patients with versus patients without behavioral disturbances. The association with cost was weaker, however, with an annual cost of $42,284 versus $32,640 per patient with versus patients without behavioral disturbances (a difference of $9644) [20]. The observed differences are likely because Aigbogun et al. had focused on specific subtypes of dementia and had only assessed the impact of behavioral disturbances; conversely, the current study examined all subtypes of dementia, including Alzheimer's disease. Our study highlights important implications for the current management strategies for people with dementia having behavioral symptoms. Inpatient costs were the biggest category of total annual costs among people with dementia and were significantly higher among those having behavioral symptoms. Length of inpatient stays was also significantly higher among people with dementia having behavioral symptoms than those without. Therefore, there is a need for timely diagnosis, appropriate medications, and management strategies to help people with dementia manage their behavioral symptoms better and potentially avert higher healthcare costs and utilization.

The current study has some shortcomings. The focus of this study were dementia patients with prevalent behavioral symptoms diagnosis. Retrospective observational studies do not provide strong causal inferences. Thus, it is challenging to ascertain whether the increased care costs identified are attributable to behavioral symptoms, mainly because the data used lack relevant information about disease progression and disease stage, which are major predictors for both intensity and frequency of behavioral symptoms. Nevertheless, our comparisons were adjusted for several patient characteristics, including drug utilization and comorbidities. There are also inherent limitations of claims data analysis. Claims are designed to track providers’ health care services for administrative purposes, namely, reimbursement. Therefore, the presence of a diagnostic code associated with a medical claim is not definitive evidence of a medical condition, intervention, or procedure. Coding errors and codes representing work performed by a provider to rule out a particular diagnosis are expected in administrative claims data and may lead to the misclassification of disease status. Furthermore, the database contains only claims records from Medicare supplemental and commercial insurance. Therefore, patients with moderate-to-severe dementia and Alzheimer’s disease who are likely to be covered by Medicare alone or dual beneficiaries covered by both Medicare and Medicaid may not be included. There is a knowledge gap regarding the validation of administrative codes for behavioral symptoms of dementia. Data limitations regarding the cost component may also exist, which may not always be reflected accurately. For instance, if Medicare is the "only" payer for any service, the claims may not be captured. In addition, health plan variations may result in some services not being reimbursed. Therefore, the cost estimate in this study may be underestimated. Lastly, this is a cross-sectional study and cannot establish causality between the presence of behavioral symptom diagnosis and elevated HCRU and costs. Even though we have accounted for the sociodemographic and extensive clinical characteristics while estimating this association, residual confounding could exist.

## Conclusion

HCRU and related costs were associated with diagnosed behavioral symptoms among patients with dementia. Further research is warranted to address the unmet medical needs of this patient population.

## Supplementary Information

**Additional file1: Supplementary Table 1.** ICD-10 Diagnostic Codes for Dementia [24].

**Additional file 2: Supplementary Table 2.** ICD-10 Diagnostic Codes for Behavioral Disturbances [24].

## Data Availability

The data supporting this study’s findings are available from the IBM® MarketScan® Commercial Claims and Encounters database. However, restrictions apply concerning their availability, which was used under license for the current research and is not publicly available. However, data are available from the corresponding author upon reasonable request and with permission from IBM® Watson Health™.

## Abbreviations

ASH: Anxiolytic, Sedative, and Hypnotic
CI: Confidence Interval
ER: Emergency Room
HCRU: Health Care Resource Utilization
ICD: International Classification of Disease
IRR: Incidence Rate Ratio
OR: Odds Ratio
U.S.: United States
